# Gut Microbiota as a Prospective Therapeutic Target for Curcumin: A Review of Mutual Influence

**DOI:** 10.1155/2018/1367984

**Published:** 2018-12-16

**Authors:** Wissam Zam

**Affiliations:** Department of Analytical and Food Chemistry, Faculty of Pharmacy, Al-Andalus University for Medical Sciences, Tartous, Syria

## Abstract

**Background:**

Turmeric is a spice that has recently received much interest and has been widely used in Ayurvedic medicine. Turmeric products are diarylheptanoids and have been characterized as safe. They are termed as curcuminoids that consists essentially of three major compounds: curcumin, demethoxycurcumin, and bisdemethoxycurcumin. Curcumin is a lipophilic polyphenol that has poor systemic bioavailability and suffers from biotransformation by human intestinal microflora to yield different metabolites that are easily conjugated to glucuronides and sulfate O-conjugated derivatives. Recently, an increasing number of studies have indicated that dysbiosis is linked with many metabolic diseases, though gut microbiota could be a novel potential therapeutic target.

**Scope and Approach:**

Thus, it is suspected that curcumin and its derivatives exert direct regulative effects on the gut microbiota which could explain the paradox between curcumin's poor systemic bioavailability and its widely reported pharmacological activities.

**Key Findings and Conclusions:**

This article summarizes a range of studies that highlight the interaction between curcumin and gut microbiota and considers opportunities for microbiome-targeting therapies using turmeric extract.

## 1. Introduction

*Curcuma longa* (turmeric) and more specifically curcumin, the main constituent of turmeric, are receiving increased clinical attention globally due to raising evidences on their therapeutic potential effects [1]. It has been demonstrated that curcumin and the whole turmeric rhizome has some potential effects in the context of chronic disease such as gastrointestinal and neurological disorders, diabetes, and cancer [2–4]. These health effects may be promoted despite low absorption by modulating intestinal barrier function [5]. Such barrier effects will in turn promote changes in the composition and diversity of the gut microbiota [5].

This article summarizes a range of studies that highlight the interaction between curcumin and gut microbiota and considers opportunities for microbiome-targeting therapies using turmeric extract.

## 2. Curcumin

**Figure 1:**
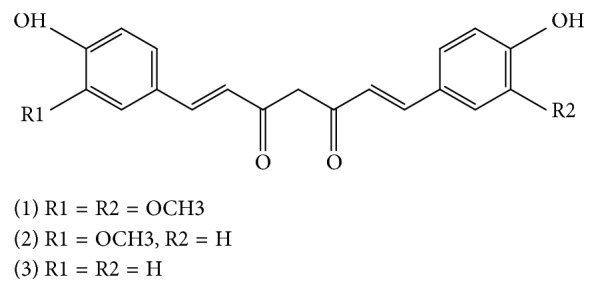
Structures of (1) curcumin (diferuloylmethane), (2) demethoxycurcumin, and (3) bisdemethoxycurcumin.

Curcumin is the product obtained by solvent extraction of turmeric and purification of the extract by crystallization. Turmeric is a spice cultivated in India and other parts of Southeast Asia and used in curries and mustards. It is a rhizomatous herbaceous perennial plant (*Curcuma longa*) of the ginger family (Zingiberaceae) that has received much interest from the culinary world as well as from the medical and scientific worlds [6, 7]. Turmeric products have been characterized as safe by several committees including the Food and Drug Administration (FDA) in the USA, the Joint Expert Committee of the Food and Agriculture Organization/World Health Organization (FAO/WHO), the Natural Health Products Directorate of Canada, and the Codex Alimentarius [8, 9]. Major phytoconstituents of turmeric are diarylheptanoids, which occur in a mixture termed curcuminoids that consists of two methoxylated phenols connected by two *α*, *β* unsaturated carbonyl groups that exist in a stable enol form, and they generally make up approximately 1−6% of turmeric by dry weight [10, 11]. The product consists essentially of three major compounds (Figure 1): curcumin (1,7-bis(4-hydroxy-3-methoxyphenyl)-1,6-heptadiene-3,5-dione, typically 60−70% of a crude extract), demethoxycurcumin (1-(4-hydroxyphenyl)-7-(4-hydroxy-3-methoxyphenyl)-hepta-1,6-diene-3,5-dione, 20−27%), and bisdemethoxycurcumin (1,7-bis-(4-hydroxyphenyl)-hepta-1,6-diene-3,5-dione, 10−15%) [6]. Curcuminoids are also reported from more than 120 Curcuma plants such as *C. phaeocaulis*, *C. aromatica*, *C. xanthorrhiza*, *C. zedoaria*, and *C. mangga* [12]. Curcumin is a lipophilic polyphenol that is nearly insoluble in water but is readily soluble in organic solvents such as acetone, dimethyl sulfoxide, and ethanol [13]. It is quite stable in the acidic pH of the stomach [14].

Turmeric is widely employed as a flavoring and coloring agent in food. Besides, it has also been widely used for its pharmacological effects in Ayurvedic medicine including antioxidant [15], analgesic, antiseptic, antispasmodic [16], antimicrobial [17, 18], anti-inflammatory [19, 20], and anticarcinogenic properties [21]. Curcumin has been consumed as a dietary supplement for centuries and is considered pharmacologically safe based on repeated studies [7]. US FDA added turmeric to the Generally Recognized As Safe (GRAS) list, and an acceptable daily intake level of 0.1–3 mg/kg-BW has been granted to curcumin by the Joint FAO/WHO Expert Committee on Food Additives, 1996 [22]. Lao et al. studied the safety of curcumin in 24 healthy volunteers using curcumin capsules with single escalating doses from 500 mg to 12,000 mg. Seven patients developed some first-grade adverse effects, including headaches, rashes, diarrhea, and yellowish stools [23].

Due to its increasing use in dietary supplements, researchers are developing many extraction methods for improving the extraction yield of curcumin. Solvent extraction followed by column chromatography is widely used for the extraction and purification of curcumin. Various methods used for extraction including soxhlet extraction, ultrasonic extraction, zone-refining, microwave, supercritical carbon dioxide, and dipping methodshave been tried [24–28].

The poor bioavailability is still one of the major problems facing the use of curcumin despite its reported benefits [29], which appear to be primarily due to poor absorption, rapid metabolism, and rapid excretion. An oral dose of 1,000 mg/kg of curcumin administered to rat resulted in approximately 75% of the dose being excreted in feces, and negligible amounts were detected in the urine [30]. Large quantities of curcumin and its metabolites were excreted in the bile of rats after intravenous and intraperitoneal administration, mainly as tetrahydrocurcumin and hexahydrocurcumin glucuronides [31]. Researchers extended their work to investigate the metabolism of curcumin using suspensions of isolated human liver or gut microsomes, and the results suggested that the metabolic reduction occurred very rapidly within minutes [32].

## 3. Gut Microbiota

All human mucosal surfaces are associated with a diverse microbial community composed mainly of bacteria but also include viruses, fungi, archaea, and protozoa [33]. The exceptionally complicated and abundant microbial community inhabits the GI tract, with 100 trillion bacteria which are remarkably 10–100 times more than the quantity of eukaryotic cells [34]. The gut environment differs markedly between different anatomical regions in terms of physiology, substrate availability, digesta flow rates, host secretions, oxygen tension, and pH [35, 36]. The large intestine is colonized by the largest obligate anaerobes microbial community due to its slow flow rates and neutral to mildly acidic pH [35, 36]. In comparison, the small intestine with its short transit times (3–5 h) and high bile concentrations provide a more challenging environment for microbial colonizers [35, 36]. Gram-positive *streptococci*, *lactobacilli*, and *enterococci* species and Gram-negative *Proteobacteria* and *Bacteroides* are the main facultative anaerobes residing in the jejunum and ileum as revealed by molecular analysis [35, 36]. Most recently, new technologies were developed, and 900 reference bacterial genome sequences were added by the Human Microbiome Project in order to assess the microbiota composition [37, 38].

The gut microbiota performs a number of essential structural, metabolic, and protective functions for host health as well as a direct action on the gut mucosa, the enteric nervous system, and far beyond the local GI compartment [39–41]. Thus, the gut microbiota resembles an endocrine organ that produces hundreds of products unlike other endocrine systems which secrete a single or at most a small number of humoral agents [42, 43]. This biochemical capacity arises from the vast and diverse array of microbial cells, with an approximate weight of 1 to 2 kg in an average adult [44].

The disturbance of this complex dual effect between gut microbiota and the host could possibly cause or contribute to disease. Accordingly, researchers are greatly interested in the diagnostic of alterations in the microbial ecology of the gut which could open new approaches in preventing or treating disease through the manipulation of the microbial gut community.

### 3.1. Dysbiosis of the Gut Microbiota in Disease

#### 3.1.1. Gut Dysbiosis

The alteration in the composition of the gut microbiota is known as gut dysbiosis and can result from exposure to various environmental factors, including diet, drugs specially antibiotics, toxins, pathogens, and increased stress [45].

The alteration in microbiota may explain why some individuals have greater risk to develop certain diseases [46]. Studies using germ-free mouse models gave the strongest evidence of the direct involvement for the gut microbiota in disease pathogenesis and it was proved that under germ-free conditions, the incidence and the severity of disease is reduced consistent with the microbiota being a “trigger” for disease progression [46].

**Figure 2:**
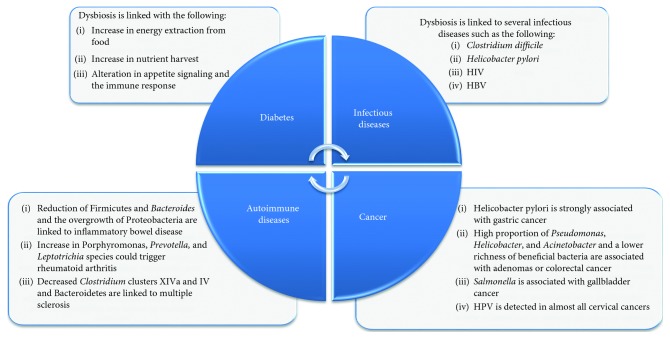
The impact of gut dysbiosis on diseases.

Various homeostatic functions of the human body could be distributed due to gut dysbiosis and this is increasingly linked to several non-communicable diseases including infectious diseases, diabetes [47], obesity [48], cancer [49], allergic asthma [50], autoimmune diseases [51], and others as presented in Figure 2.

Several studies have demonstrated an important relationship between infection and dysbiosis [52] such as the infection with *Clostridium difficile* [53] and *Helicobacter pylori* [54]. Results also showed that infection is associated not only with the microbiome, but also with viruses [55] such as human immunodeficiency virus (HIV) [56] and hepatitis B virus (HBV) [57].

An increasing number of studies have indicated a great interaction between the gut microbiota dysbiosis and several metabolic disorders including obesity and diabetes [58, 59]. Germ-free mice have reduced adiposity and improved tolerance to glucose and insulin when compared with conventional counterparts when fed a Western-style diet [60]. Increased adiposity was observed in lean mice after receiving a microbiota transplant from genetically obese mice characterized by an altered microbiota [61]. These interactions are mediated via several mechanisms including the potential to increase nutrient harvest and energy extraction from food; and alter appetite signaling and the immune response [62, 63].

The relationship between human carcinogenesis and specific pathogenic bacteria has been widely investigated. Multiple studies revealed that individuals diagnosed with gastrointestinal malignancy have different gut microbiome composition compared with healthy individuals. The chronic inflammation caused by *Helicobacter pylori* is considered to be the strongest risk factor for gastric cancer and its eradication before the onset of chronic atrophic gastritis may protect against gastric cancer [63]. Beyond *H. pylori*, the synergetic colonization of altered Schaedler's flora causes gastric corpus inflammation, epithelial hyperplasia, and dysplasia in insulin-gastrin mice [64]. The effect of the gut microbiome in the development and progress of colorectal cancer has recently become a major focus of research. An increase in adenomas or colorectal cancer is observed in subjects with a high proportion of potential pathogens, such as *Helicobacter*, *Pseudomonas*, and *Acinetobacter*, and a lower richness of beneficial bacteria especially butyrate-producing bacteria [65]. A significant increase in *Bacteroides massiliensis*, *Bacteroides vulgatus*, *Bacteroides ovatus*, *Fusobacterium nucleatum*, and *E. coli* has also been observed from advanced adenoma to carcinoma [66, 67]. Sharma et al. showed an association between *Salmonella* and gallbladder cancer [68]. Cancer risk is also influenced by viruses which are also a component of the gut microbiome. For example, DNA from human papillomavirus (HPV) is detected in almost all cervical cancers [69].

On the contrary, accumulating evidence indicates that the therapeutic activity and the side effects of anticancer agents administered orally or parenterally could both be influenced by the gut microbiota via pharmacodynamics and immunological mechanisms [70].

Several gut microbiota mechanisms are involved in the promotion of autoimmunity. It is hypothesized that an aberrant modification of host proteins could be due to the changed spectrum of microbial enzymes involved in posttranslational modification of proteins (PTMP) which may contribute to autoimmune diseases by generating autoimmune responses [71]. Under the germ-free conditions, no autoimmune disease is developing in the animal models, while some bacterial species are directly linked to the progression of specific autoimmune diseases [72]. Reduction of *Firmicutes* and *Bacteroides* and the overgrowth of *Proteobacteria* are linked to inflammatory bowel disease [72]. Increasement in *Porphyromonas, Prevotella*, and *Leptotricha* species could trigger rheumatoid arthritis [73]. Decreased *Clostridia* clusters XIVa and IV and *Bacteroidetes* are linked to multiple scelorsis [74].

### 3.2. Life Style and Dietary Effect on Gut Microbiota

Smoking, stress, and lack of exercise can greatly impact the gut microbiota composition. Indeed, smoking has a great impact on gut microbiota composition by increasing *Bacteroides-Prevotella* [75]. Stress has a significant influence on colonic motor activity via the gut-brain axis involving both hormonal and neuronal pathways. This impact is associated with an altered gut microbiota profiles, including a decrease in numbers of potentially beneficial *Lactobacillus* [41, 76].

Protein, carbohydrates, and fat are the most comment and major components in diets of human that have been widely found to impact the composition of the gut microbiota in the host. The end products of protein degradation at the distal end of the colon are amino acids, amines, ammonia, and SCFA. A diet containing a high concentration of cysteine or threonine can cause a significant increase in beneficial microbiota such as *lactobacilli* or *bifidobacteria* and a decrease in *Clostridiaceae* [77]. Complex carbohydrates such as insulin and oligosaccharides, also referred to as prebiotics, can be degraded by proteolytic enzymes into short chain fatty acids and various gases and are normally an important energy resource for microbial growth. Prebiotics also act as important stimulants which promote the growth of beneficial bacteria such as *bifidobacteria* and *lactobacilli* [78]. The consumption of high-fat foods tends to induce substantial alterations in the composition of GI tract microbiota by increasing *Rikenellaceae* and decreasing *Ruminococcaceae* [79].

Habitual dietary pattern and shorter term dietary variation influences gut microbiota composition at the genus and species level. Western diet characterized by a high proportion of total and saturated fats, animal protein, and simple sugars with a low proportion of plant-based foods, is associated with gut microbial populations that are typified by a *Bacteroides* enterotype. In contrast, plant based diets containing a high proportion of polysaccharides are associated with a *Prevotella* enterotype known to use cellulose and xylans as substrates [80, 81] with a greater diversity of the fecal microbiota compared with individuals consuming habitual Western diets [82].

Rapid and marked alterations in fecal microbiota composition especially in *Bacteroides* to *Prevotella* ratio are observed when replacing a habitual Western diet with one high in fiber can cause [80]. The Mediterranean diet based on fruits and vegetables, monounsaturated and polyunsaturated fats and grains, is considered as a standard diet for a healthy life style. Individuals fed on the Mediterranean diet have lower numbers of *Bacillaceae* and *Proteobacteria* but higher *Clostridium* and *Bacteroidetes* populations [83]. Additionally, vegetarian diets could decrease the ratio of *Clostridium cluster* XIVa species, but increase the number of *Faecalibacterium prausnitzii*, *Clostridium clostridioforme*, and *Bacteroides Prevotella* [84].

## 4. Effects of Curcumin on Gut Microbiota

Aside of the poor systemic bioavailability of curcumin, it is expected to find it at high concentrations in the gastrointestinal tract after oral administration. Thus, it is suspected that curcumin could exert direct regulative effects on the gut microbiota which could explain the paradox between curcumin's poor systemic bioavailability and its widely reported pharmacological effects, as resumed in Table 1 [85]. The administration of curcumin significantly shifted the ratio between beneficial and pathogenic microbiota by increasing the abundance of *bifidobacteria, lactobacilli*, and butyrate-producing bacteria and reducing the loads of *Prevotellaceae*, *Coriobacterales*, *enterobacteria*, and *enterococci*. These alterations in gut microbiota could explain the immune modulation and antihyperlipidemia efficacy of curcumin aside of its anti-inflammatory and anticolonotropic carcinogenicity activity.

Shen et al. [86] investigated the regulative effects of oral curcumin administration of 100 mg/kg body weight on the gut microbiota of C57BL/6 mice. After 15 days of continuous once daily oral dose of curcumin, a total of 370 shared operational taxonomic units (OTUs) between the curcumin and control groups, and 39 were unique in the curcumin group and 79 in the control group. Curcumin was found to decrease the microbial richness and diversity, with significant differences in abundance between the curcumin and control groups in three bacterial families [86]. A significant decrease in the abundance of *Prevotellaceae* was observed, while the abundance of *Bacteroidaceae* and *Rikenellaceae* was significantly increased in the curcumin group [86]. *Prevotella* species are anaerobic Gram-negative bacteria of the *Bacteroidetes* phylum that were found to be greater in CRC patients than in stool from cancer-free patients [87]. The role of *Prevotella* in driving Th17-mediated immune responses in periodontitis is clarified by results of studies that found a significant link between IL-1a and IL-1b levels in crevicular fluid and *Prevotella* colonization [88].

In order to study the effect of curcumin-supplemented diet on colonotropic carcinogenicity, mice received intraperitoneal injections of the mutagenic agent azoxymethane. A relative increase in the abundance of *Lactobacillales* and a decrease in *Coriobacterales* order was observed with a curcumin-supplemented, and this effect was correlated with entire eliminated tumor burden [89]. A large systemic review summarizes the original articles studying the relation between microbiota and colorectal cancer until November 2014. It showed that some bacteria are consistently diminished in colorectal cancer such as *Bifidobacterium*, *Lactobacillus*, *Ruminococcus,* and *Faecalibacterium* spp, while others are constantly augmented such as *Coriobacteridae*. It is also clear that bacteria metabolites amino acids are increased and butyrate is decreased throughout colonic carcinogenesis [90]. Preclinical studies have consistently shown that curcumin possesses anticancer activity *in vitro* and in preclinical animal models via the activation of caspases 9, 3, and 8 in the colon cancer cell lines SW480 and SW620 [91]. In four colon cancer cell lines (HT-29, IEC-18-k-ras, Caco-2, and SW-480), the use of celecoxib (5 lM) and curcumin (10–15 lM) inhibited the proliferation and induced apoptosis through the COX-2 and non-COX-2 pathways [92]. Recently, a number of studies have suggested that curcumin has the potential to target cancer stem cells (CSC) through direct or indirect influences on the CSC self-renewal pathways [93]. Its robust activity in colorectal cancer has led to five phase I clinical trials being completed showing the safety and tolerability of curcumin in colorectal cancer patients using doses up to 8000 mg per day [94, 95]. The success of these trials has led to the development of phase II trials that are currently enrolling patients [96].

In another study, the effects of nanoparticle curcumin on experimental colitis in mice via the modulation of gut microbiota were studied [97]. BALB/c mice were fed with 3% dextran sulfate sodium in water. Treatment with nanoparticle curcumin suppressed mucosal mRNA expression of inflammatory mediators and the activation of NF-κB in colonic epithelial cells. These effects were accompanied with an increase in the abundance of butyrate-producing bacteria and fecal butyrate level [97].

Previous studies in active IBD and in experimental DSS-colitis [98, 99] have shown that curcumin can ameliorate intestinal inflammation through modulation of intracellular signaling transduction pathways and different molecular pathways including immunoregulatory and anti-inflammatory mechanisms [100, 101]. A preclinical study found an antiatherogenic effect of low dose of curcumin in a mouse model of atherosclerosis [102].

It was found that curcumin attenuates Western diet-induced development of type 2 diabetes mellitus and atherosclerosis [103]. This could be explained by the efficacy of curcumin on reversing the effect of high-fat diet on the composition of the gut microbiota by shifting it toward that of the lean comparison rats fed a normal diet [104]. The anti-inflammatory effects of curcumin were studied in animal models infected with *Toxoplasma gondii*. It was found that curcumin-supplemented animals showed fewer proinflammatory *enterobacteria* and *enterococci* and higher anti-inflammatory *bifidobacteria* and *lactobacilli* loads [105]. It was found that low doses of curcumin attenuate diet-induced hypercholesterolemia in rats and boosted high-density lipoprotein cholesterol levels [106, 107].

Estrogen deficiency induced by ovariectomy caused alterations in the structure and distribution of intestinal microflora in rats, and the administration of curcumin could partially reverse changes in the diversity of gut microbiota according to Zhang et al. [108]. The effects of curcumin on gut microfloral communities of ovariectomized (OVX) female rats were studied, and the results indicated that gut microbiota of rats from the curcumin-treated group (CUR) had higher levels of biodiversity and unevenness estimations than those from the OVX group [108]. Seven differential gut microbiota (*Anaerotruncus*, *Exiguobacterium*, *Helicobacter, Papillibacter*, *Pseudomonas*, *Serratia*, and *Shewanella*) between OVX and CUR groups were found [108].

Dey et al. transplanted six groups of gnotobiotic mice with fecal microbes derived from one of six healthy adults with various ethnic dietary patterns. The results of this study provided evidence on the impact of regional diets on microbiota function [109]. The authors report that turmeric altered microbiome composition and function, slowed transit by altering bile acid metabolism, and affected intestinal motility [109].

In a more recent study, Peterson et al. investigated the effects of turmeric and curcumin dietary supplementation on human gut microbiota by a double-blind, randomized, placebo-controlled pilot study [110]. Turmeric tablets with extract of piperine (Bioperine), curcumin with Bioperine tablets, or placebo tablets were provided to healthy human subjects and subsequent changes in the gut microbiota were determined by 16S rDNA sequencing. The results indicated a significant and individualized variation in gut microbiota over time. Turmeric and curcumin treatment resulted in the reduced average relative abundance of 71 and 56 taxa, respectively [110].

The results of these various studies strongly suggest that curcumin may act as promoting factors of growth, proliferation, or survival for beneficial members of the gut microbiota. A number of mechanisms may account for the stimulatory effect of curcumin. The first proposed mechanism lies on the ability of some microorganisms to use polyphenols as substrates. Besides, phenolic compounds positively affect bacteria consumption of nutrients such as sugars. One study examined the effects of turmeric in 8 healthy human participants fasted for 12 h and ingested curry and rice with or without turmeric. Results showed that turmeric increased the AUC of breath hydrogen compared with turmeric-free diet, suggesting that dietary turmeric activated carbohydrate colonic fermentation [111].

Another proposed mechanism depends on the increased abundance of certain *lactobacilli* strains that can strongly inhibit gastrointestinal pathogens due to the production of lactic acid which influences the pathogen invasion of human epithelial cells [112]. Additionally, in the case of some strains, such as *L. johnsonii* La1 and *L. plantarum* ACA-DC 287, a combination of lactic acid and bacteriocin-like compounds production was also detected [113].

## 5. Effects of Gut Microbiota on Curcumin

The gut microbiota plays an important role in the metabolism and biotranformation of curcumin into a range of catabolites [29]. It was noticed that the biotransformation of turmeric curcuminoids by human GM is reminiscent of equal production from the soybean isoflavone daidzein [114]. Tan et al. used an *in vitro* model containing human fecal starters to investigate the colonic metabolism of curcuminoids. Results showed that after 24 h of fermentation *in vitro*, up to 24% of curcumin, 61% of demethoxycurcumin, and 87% of bisdemethoxycurcumin were degraded by the human fecal microbiota. Three main metabolites were detected in the fermentation cultures, namely, tetrahydrocurcumin (THC), dihydroferulic acid (DFA), and 1-(4-hydroxy-3-methoxyphenyl)-2-propanol [115]. Analyses of microorganisms isolated from human feces revealed that *E. coli* exhibited the highest curcumin-metabolizing activities via NADPH-dependent curcumin/dihydrocurcumin reductase [116].

It has been reported that microbial metabolism of curcumin with *Pichia anomala* yielded four major metabolites, 5-hydroxy-7-(4-hydroxy-3-methoxyphenyl)-1-(4-hydroxyphenyl)heptan-3-one, 5-hydroxy-1,7-bis(4-hydroxy-3-methoxyphenyl)heptan-3-one, 5-hydroxy-1,7-bis(4-hydroxyphenyl)heptane-3-one, 1,7-bis(4-hydroxy-3-methoxyphenyl)heptan-3,5-diol, and two minor products [117].

Li et al. proved that the curcumin metabolism in the GI tract is complicated and underwent different stages. They demonstrated that phase I metabolism yielded three metabolites, namely, tetrahydrocurcumin (M1), hexahydrocurcumin (M2), and octahydrocurcumin (M3) [118]. Then, curcumin and these phase I metabolites were subject to conjugation via phase II metabolism to yield their corresponding glucuronide and sulfate O-conjugated metabolites [119, 120]. Gut microbiota may deconjugate the phase II metabolites and convert them back to the corresponding phase I metabolites and some fission products such as ferulic acid in the cecum and colon [121].

In a recent research, the metabolic profile of curcumin in human intestinal flora was identified *in vitro* using ultraperformance liquid chromatography/quadrupole time-of-flight mass spectrometry. On the basis of the used method and the metabolites identified, reduction, methylation, demethoxylation, hydroxylation, and acetylation were the main pathways by which curcumin was metabolized by human intestinal microflora to yield 23 different metabolites [119]. Reductive metabolites are the predominant metabolites in the human intestinal microflora system and appear to be easily conjugated [120]. Glucuronidation is the dominating pathway of conjugation, and the glucuronide of hexahydrocurcumin is usually found as the major metabolite of curcumin in body fluids, cells, and organs [121].

There is evidence that curcumin metabolites display a similar potency to curcumin [122]. Tetrahydrocurcumin (THC), a major metabolite of curcumin, has been demonstrated to act against neurodegeneration, to prevent inflammation and oxidative stress, and to possess antitumor activity [123]. These effects could be due to the inhibition of prominent cytokines release, including interleukin-6 (IL-6) and tumor necrosis factor-*α* (TNF-*α*); however, octahydrocurcumin (OHC) and hexahydrocurcumin (HHC) did not significantly alter cytokine release [123]. Furthermore, LPS-mediated upregulation of iNOS and COX-2 as well as NF-*κ*B activation were significantly inhibited by the three curcumin metabolites (THC, HHC, and OHC) [124].

A bacterial strain of *Bacillus megaterium* DCMB-002, isolated from mice feces, showed the capability of transforming curcumin to seven metabolites through different metabolic processes including hydroxylation, demethylation, reduction, and demethoxylation. After 24 h of incubation, the metabolites exhibited moderate antioxidant activity [125].

## 6. Conclusions and Perspective

Both gut microbiota and diet impact each other and can strongly affect our health. The development of a rich and stable gut microbiota is crucial for maintaining proper host physiologic functions. However, dysbiosis, characterized by reduced diversity and the predominance of a few pathogenic taxa, is linked with many metabolic diseases.

Curcumin attracted researchers and has received worldwide attention for its multiple pharmacological activities, which appear to act primarily through its anti-inflammatory and antioxidant mechanisms. Given the low systemic bioavailability of curcumin and its pharmacological therapeutic uses, curcumin might provide benefit by acting on gut microbiota. This impact on the gut microbiota seems to be reasonable and attractable areas of study as no absorption of the parent compound is necessary. In addition, it was proved that the composition of gut microbiota had a profound influence on the biotransformation of curcumin in the colon by various processes mainly by reduction followed by conjugation, which might have a significant impact on the health effects of dietary curcumin, especially in the GI.

Future researches on human volunteers are required to extend the current gut microbiota outcomes in order to provide a basis for gut microbiota-based therapeutic applications of curcumin. They should also lay in an individualized approach based on a comprehensive analysis of differences in gut microbiota between individuals and their exact curcumin intake, taking into account their genetic and epigenetic predispositions.

## Figures and Tables

**Table 1:** Effects of curcumin on gut microbiota.

Dose	Effect on microbiota	Benefits and mechanism	References
100 mg/kg once daily for 15 days	A significant decrease in the abundance of *Prevotellaceae* was observed, while the abundance of *Bacteroidaceae* and *Rikenellaceae* was significantly	*Prevotella* species were found to be greater in CRC patients. The role of Prevotella in driving Th17-mediated immune responses is clarified by the increase of IL-1a and IL-1b levels in crevicular fluids	[86–88]
Curcumin-supplemented diet at doses up to 8000 mg per day	A relative increase in the abundance of *Lactobacillales* and a decrease in *Coriobacterales* order was observed	Curcumin possesses anticancer activity *in vitro* and in preclinical animal models via the activation of caspases 9, 3, and 8 in the colon cancer cell lines. It also inhibited the proliferation and induced apoptosis through the COX-2 and non-COX-2 pathways. It has also the potential to target cancer stem cells (CSC) through direct or indirect influences on the CSC self-renewal pathways.	[89–96]
0.2% (w/w) nanoparticles of curcumin	An increase in the abundance of butyrate-producing bacteria and fecal butyrate level was observed	Nanoparticles of curcumin suppressed mucosal mRNA expression of inflammatory mediators and the activation of NF-κB in colonic epithelial cells	[97]
Up to 2000 mg/day	Curcumin-supplementation showed fewer proinflammatory *enterobacteria* and *enterococci* and higher anti-inflammatory *bifidobacteria* and *lactobacilli* loads	It can ameliorate intestinal inflammation through modulation of intracellular signaling transduction pathways and different molecular pathways including immunoregulatory and anti-inflammatory mechanisms	[98–101, 105]
Low dose of curcumin (1 g/day)	An increase in the abundance of butyrate-producing bacteria	Antiatherogenic and antihypercholesterolemia effects by increasing HDL levels	[102, 106, 107]
Low dose of curcumin (1 g/day)	Curcumin supplementation shifts the composition of the gut microbiota toward that of the lean comparison rats	Curcumin attenuates Western diet-induced development of type 2 diabetes mellitus and atherosclerosis	[103, 104]
100 mg/kg/day	Curcumin could partially reverse changes in the diversity of gut microbiota in estrogen deficient rats. At the phyla level, a decrease of phyla Firmicutes and Bacteroidetes was observed.	Curcumin had a significant preventive effect on body weight gain. In addition, it decreases the estradiol serum levels.	[108]
